# Development and validation of a scoring system to predict the mortality of hospitalized patients with SARS-CoV-2 Omicron: a nationwide, multicentre study

**DOI:** 10.1186/s12890-024-03131-5

**Published:** 2024-07-03

**Authors:** Wanru Guo, Xiaomeng Li, Cheng Ding, Xiahong Dai, Shuai Wu, Yunzhen Shi, Yongjun Jiang, Yukun Chang, Zhidan Zhang, Shiyang Liu, Lei Ma, Yu Zhang, Tong Zhao, Wenjuan Hu, Jiafeng Xia, Yanwan Shangguan, Kaijin Xu

**Affiliations:** 1 State Key Laboratory for Diagnosis and Treatment of Infectious Diseases, National Clinical Research Center for Infectious Diseases, Collaborative Innovation Center for Diagnosis and Treatment of Infectious Diseases, The First Affiliated Hospital, National Medical Center for Infectious Diseases, Zhejiang University School of Medicine, Hangzhou, China; 2 Department of Infectious Diseases, Hospital Affiliated to Zhejiang Shuren University Shulan International Medical College, Shulan, Hangzhou, China; 3 Department of Infectious Diseases, Shenzhen Third People’s Hospital, Shenzhen, China; 4 Department of Infectious Diseases, Affiliated Dongyang Hospital of Wenzhou Medical University, Dongyang, Zhejiang China; 5 Key Laboratory of AIDS Immunology (China Medical University), National Clinical Research Center for Laboratory Medicine, National Health Commission (NHC), The First Hospital of China Medical University, Shenyang, China; 6 Department of Infectious Disease, The First Affiliated Hospital of China Medical University, Shenyang, China; 7 Institute of Infectious Diseases, Fifth Medical Center of People’s Liberation Army General Hospital, Beijing, China; 8 Center of Liver Diseases Division 3, Beijing Ditan Hospital, Capital Medical University, Beijing, China; 9 Department of Hepatology, Qilu Hospital of Shandong University, Ji’nan, China; 10 Infection Control Department, The First Affiliated Hospital, Zhejiang University School of Medicine, Hangzhou, China

**Keywords:** COVID-19, SARS-CoV-2 Omicron variant, Scoring system, Mortality, Multicentre study

## Abstract

**Background:**

The Omicron variant broke out in China at the end of 2022, causing a considerable number of severe cases and even deaths. The study aimed to identify risk factors for death in patients hospitalized with SARS-CoV-2 Omicron infection and to establish a scoring system for predicting mortality.

**Methods:**

1817 patients were enrolled at eight hospitals in China from December 2022 to May 2023, including 815 patients in the training group and 1002 patients in the validation group. Forty-six clinical and laboratory features were screened using LASSO regression and multivariable logistic regression.

**Results:**

In the training set, 730 patients were discharged and 85 patients died. In the validation set, 918 patients were discharged and 84 patients died. LASSO regression identified age, levels of interleukin (IL) -6, blood urea nitrogen (BUN), lactate dehydrogenase (LDH), and D-dimer; neutrophil count, neutrophil-to-lymphocyte ratio (NLR) as associated with mortality. Multivariable logistic regression analysis showed that older age, IL-6, BUN, LDH and D-dimer were significant independent risk factors. Based on these variables, a scoring system was developed with a sensitivity of 83.6% and a specificity of 83.5% in the training group, and a sensitivity of 79.8% and a sensitivity of 83.0% in the validation group.

**Conclusions:**

A scoring system based on age, IL-6, BUN, LDH and D-dime can help clinicians identify patients with poor prognosis early.

**Supplementary Information:**

The online version contains supplementary material available at 10.1186/s12890-024-03131-5.

## Background

Coronavirus disease (COVID-19), caused by severe acute respiratory syndrome coronavirus 2 (SARS-CoV-2), has affected almost all countries and regions, posing a great threat to human health. SARS-CoV-2 has evolved into various variants with different virulence and transmission, including Alpha (B.1.1.7), Beta (B.1.351), Gamma (P.1), Delta (B.1.617.2) and Omicron (B.1.1.529) [1]. B.1.1.529 was first discovered in South Africa in November 2021, and was listed as a Variants of Concern by the World Health Organization and named Omicron [2]. Increased transmissibility and reduced protection from neutralising antibodies have led to the rapid spread of this variant, which rapidly became a major variant in many countries [3]. Since the cancellation of the zero-COVID policy in China in December 2022, many cases of SARS-CoV-2 Omicron infection have been reported across the country [4]. The clinical manifestations of Omicron infection vary widely, ranging from asymptomatic illness to pneumonia and life-threatening complications, including acute respiratory distress syndrome and multiple organ failure, and death [5]. Although the case fatality rate is lower than that of ancestral viral strains, the risk of death remains high in older age groups, particularly in patients with comorbidities. Identifying patients with a poor prognosis early and providing supportive treatment are important for improving prognosis. The development of prediction models can provide a basis for medical decisions and help medical workers manage patients with different risks better.

Previously reported poor prognostic factors for COVID-19 include advanced age, multiple comorbidities, low lymphocyte count, elevated levels of inflammatory markers, coagulation markers, and cytokines, and imaging features [6, 7]. Cytokine storms are associated with severe COVID-19, and many studies have reported that proinflammatory cytokines, such as interleukin (IL)-1, IL-6, and tumour necrosis factor, can be used as prognostic biomarkers [8, 9]. Although several models have been developed for predicting severe disease and death in patients with COVID-19, most of the existing prediction models have defects such as complex calculation methods, high risk of bias, and lack of multicentre data verification, and are unsuitable for clinical application. Considering the differences in epidemiology and clinical characteristics between Omicron and other earlier variants, and paucity of reports on high-quality prediction models related to severe Omicron infection, we systematically studied the prognostic biomarkers of mortality in patients with SARS-CoV-2 Omicron infection, developed a predictive scoring system, and validated it using a national multicentre data to predict the risk of death in hospitalized patients with SARS-CoV-2 Omicron infection.

## Methods

### Study design and population

This retrospective multicentre study included data on 1817 patients hospitalized with SARS-CoV-2 Omicron infection at eight hospitals in China between December 19, 2022 and May 25, 2023. All enrolled patients were hospitalized for COVID-19. The diagnostic criteria were as follows: (i) clinical manifestations associated with COVID-19; (ii) a positive SARS-CoV-2 nucleic acid or antigen test result and (iii) a clear treatment outcome of discharge or death. Exclusion criteria were (i) age less than 14 years old, (ii) still receiving treatment at the time of data analysis or (iii) lack of information on underlying diseases. The clinical outcomes such as discharges or mortality were monitored up to June 10, 2023. The 815 patients from the First Affiliated Hospital of Zhejiang University constituted the training set, and the remaining 1002 patients from seven hospitals, including Shenzhen Third People’s Hospital, The First Affiliated Hospital of China Medical University, Affiliated Dongyang Hospital of Wenzhou Medical University, Shulan Hospital of Hangzhou, Fifth Medical Center of People’s Liberation Army General Hospital, Beijing Ditan Hospital Affiliated to Capital Medical University, and Qilu Hospital of Shandong University, constituted the external validation set.

### Data collection

Baseline demographic characteristics (age and sex), clinical data (onset of symptoms, underlying diseases and laboratory test results on admission) and treatment outcomes were obtained from the electronic medical record system. Laboratory tests included haematology, serum biochemistry, coagulation spectrum, infection-related factors and cytokine levels. Data were collected on the time from onset to admission, and time from admission to discharge or death.

### Statistical analysis

Categorical variables were described using frequencies and percentages, and continuous variables were described using medians and interquartile ranges (IQRs). The Mann-Whitney U-test was used to compare continuous variables, and the chi-square test or Fisher’s exact test was used to compare categorical variables. Continuous variables were converted into binary variables according to the cutoff value determined using the receiver operating characteristic (ROC) curve, with optimal sensitivity and specificity. Least absolute shrinkage and selection operator (LASSO) regression analysis was performed to identify variables with non-zero coefficients using the R “glmnet” software application [10]. Forty-six clinical features with missing values < 10% were included in the variable shrinkage process. A logistic risk model was used to establish the LASSO regression, and the optimal lambda value with the smallest partial likelihood deviation was selected using a 10-fold cross-validation. The variables screened by LASSO regression were further analysed using multivariable logistic regression analysis. To develop prognostic scores, we used the regression coefficients of prognostically relevant variables and assigned points proportional to the coefficient. To quantify the discriminant performance of the model, ROC curve and area under the curve (AUC) analysis were performed using the R “pROC” software package. All analyses were performed using R software (version 3.6.3). P values < 0.05 were considered statistically significant.

## Results

### Patient demographic and clinical characteristics

The training set included 815 patients, of whom 85(10.4%) died and 730 (89.6%) survived (Table 1). The median age of the patients was 72 (IQR, 61–82) years, with 500 males (61.3%). The median interval from onset to admission was 8 (IQR, 6–10) days, and the median length of hospitalisation was 8 (IQR, 6–10) days. The validation set included 1002 patients, of whom 84 (8.4%) died and 918 survived. The median age of the patients was 62 (IQR, 47–76) years, with 630 males (62.9%). The median interval from onset to admission was 2.5 (IQR, 1–7) days, and the median length of hospitalisation was 12 (IQR, 7–17) days. In both sets, the top three comorbidities were hypertension, diabetes, cardio-cerebrovascular disease (CCVD), and the most common symptoms were fever and cough. Compared with survivors, non-survivors were older, with a significantly higher prevalence of dyspnoea and comorbidities (hypertension, diabetes, and CCVD), and a longer length of hospital stay. Continuous variables were converted to binary variables based on cutoff values determined by the receiver operating characteristic (ROC) curve for optimal sensitivity and specificity. The cutoff values were described in Tables 1 and 2. A comparison of the laboratory test results on admission of survivors and non-survivors in the training set is shown in Table 2.

**Table 1:** Demographic and clinical characteristics of the training set and validation set on admission

Characteristics	Training set (*n* = 815)					External validation set (*n* = 1002)			
	all	Survivors (*n* = 730)	Non-survivors (*n* = 85)	*p*-value		all	Survivors (*n* = 918)	No-survivors (*n* = 84)	*p*-value
Age, years, median (IQR)	72(61–82)	71(60–81)	82(74–89)	**< 0.001** ^*^		62(42–76)	60(40–74)	80(70–86)	**< 0.001** ^*^
≥ 78 y, n(%)	292(35.8%)	236(32.3%)	56(65.9%)	**< 0.001** ^*^		228(22.8%)	183(19.9%)	45(5.4%)	**< 0.001** ^*^
Males, n(%)	500(61.3%)	447(61.2%)	53(62.4%)	0.934		630(62.9%)	565(61.5%)	65(77.4%)	**0.006** ^*^
Days from illness onset to Hospital admission, median (IQR)	8(6–10)	8(6–10)	7(4–10)	**0.012** ^*^		2.5(1–7)	2(1–7)	5.5(3–10)	**< 0.001** ^*^
Days from hospital admission to outcome, median (IQR)	8(6–13)	8(6–12)	14(8–23)	**< 0.001** ^*^		12(7–17)	11(7–16)	15(9–25)	**0.001** ^*^
**Comorbidity**									
Hypertension, n(%)	416(51.0%)	358(49.0%)	58(68.2%)	**0.001** ^*^		356(35.5%)	307(33.4%)	49(58.3%)	**< 0.001** ^*^
Diabetes, n(%)	187(22.9%)	158(21.6%)	29(34.1%)	**0.014** ^*^		206(20.6%)	180(19.6%)	26(31%)	**0.02** ^*^
Chronic obstructive pulmonary disease, n(%)	73(9.0%)	68(9.3%)	5(5.9%)	0.396		84(8.4%)	74(8.1%)	10(11.9%)	0.312
Cardio-cerebral-vascular disease, n(%)	132(16.2%)	109(14.9%)	23(27.1%)	**0.007** ^*^		229(22.9%)	191(20.8%)	38(45.2%)	**< 0.001** ^*^
Chronic liver disease, n(%)	21(2.6%)	17(2.3%)	4(4.7%)	0.263		108(10.8%)	103(11.2%)	5(6%)	0.5
Chronic kidney disease, n(%)	94(11.5%)	80(11%)	14(16.5%)	0.185		112(11.2%)	94(10.2%)	18(21.4%)	**0.003** ^*^
Malignant tumor, n(%)	78(9.6%)	68(9.3%)	10(11.8%)	0.595		140(14.0%)	130(14.2%)	10(11.9%)	0.684
**Symptoms**									
Fever, n(%)	674(82.7%)	608(83.3%)	66(77.6%)	0.25		599(59.8%)	533(58.1%)	66(78.6%)	**< 0.001** ^*^
Cough, n(%)	706(86.6%)	638(87.4%)	68(80%)	0.084		559(55.8%)	504(54.9%)	55(65.5%)	0.080
Dyspnea, n(%)	493(60.5%)	429(58.8%)	64(75.3%)	**0.005** ^*^		232(23.2%)	187(20.4%)	45(53.6%)	**< 0.001** ^*^
Fatigue, n(%)	226(27.7%)	201(27.5%)	25(29.4%)	0.812		239(23.9%)	218(23.7%)	21(25%)	0.901
Nausea/vomiting, n(%)	47(5.8%)	42(5.8%)	5(5.9%)	1		38(3.8%)	34(3.7%)	4(4.8%)	0.552
Diarrhea, n(%)	31(3.8%)	28(3.8%)	3(3.5%)	1		43(4.3%)	39(4.2%)	4(4.8%)	0.777

*Notes* *IQR* Interquartile Range; Statistical significance is indicated in bold

**Table 2:** Laboratory test results of patients in the training set on admission

Laboratory indexes	All (*n* = 815)	Survivors (*n* = 730)	Non-survivors (*n* = 85)	*P* value
**Routine peripheral blood**				
WBC (×10^9^/L), median (IQR)	6.04 (4.30–8.81)	5.86 (4.1–8.33)	9.11 (5.99–11.6)	**< 0.001** ^*^
WBC > 7.4 × 10^9^/L, n (%)	280 (34.4)	226 (31.0)	54 (63.5)	**< 0.001** ^*^
Neu (×10^9^/L), median (IQR)	4.78 (3.07–7.33)	4.54 (2.94–6.59)	7.93 (4.86–10.6)	**< 0.001** ^*^
Neu > 7 × 109/L, n (%)	219 (26.9)	167 (22.9)	52 (61.2)	< 0.001*
Lym (×109/L), median (IQR)	0.72 (0.48–1.05)	0.76 (0.5–1.1)	0.5 (0.3–0.65)	< 0.001*
Lym > 0.7 × 10^9^/L, n (%)	388 (47.6)	321 (44.0)	67 (78.8)	**< 0.001** ^*^
NLR, median (IQR)	6.57 (3.38–11.75)	5.87 (3.21–10.2)	16.1 (8.41–26)	**< 0.001** ^*^
NLR > 8, n (%)	336 (41.2)	267 (36.6)	69 (81.2)	**< 0.001** ^*^
PLT(×10^9^/L), median (IQR)	190 (141.5–245)	191 (145–246)	172 (118–232)	**0.018**
PLT < 149 × 10^9^/L, n (%)	233 (28.6)	196 (26.8)	37 (43.5)	**0.002**
**Blood biochemical examination**				
ALT(U/L), median (IQR)	21 (14–33)	20.5 (14–33)	22 (12–34)	0.881
ALT > 23 U/L, n (%)	351 (43.1)	310 (42.5)	41 (48.2)	0.368
AST (U/L), median (IQR)	24 (18–36)	24 (18–34)	31 (20–47)	**< 0.001** ^*^
AST > 33 U/L, n (%)	250 (30.7)	211 (28.9)	39 (45.9)	**0.002**
Albumin (g/L), median (IQR)	40.7 (34.6–60.8)	41 (34.9–61)	38.3 (31.7–57.9)	**0.011**
Albumin > 34 g/L, n (%)	181 (22.2)	148 (20.3)	33 (38.8)	< 0.001*
TBIL (umol/L), median (IQR)	7.7 (5.5–10.45)	7.5 (5.4–103)	8.4 (5.8–12)	**0.037**
TBIL > 10 umol/L, n (%)	229 (28.1)	195 (26.7)	34 (40.0)	**0.014**
DBIL (umol/L), median (IQR)	6.52 (4.89–9.24)	3.7 (2.7–5.2)	4.7 (3–8.4)	**< 0.001** ^*^
DBIL > 4 umol/L, n (%)	372 (45.6)	317 (43.4)	55 (64.7)	**< 0.001** ^*^
Cr (umol/L), median (IQR)	76 (61–97)	74 (60–92)	96 (69–196)	**< 0.001** ^*^
Cr > 95 umol/L, n (%)	216 (26.5)	169 (23.2)	47 (55.3)	**< 0.001** ^*^
TG(mmol/L), median (IQR)	1.04 (0.8–1.37)	1.04 (0.8–1.36)	1.18 (0.85–1.48)	**0.02**
TG > 1.2 mmol/L, n (%)	286 (35.1)	251 (34.4)	35 (41.2)	0.262
BUN(mmol/L), median (IQR)	6.52 (4.89–9.24)	6.21 (4.78–8.5)	11.1 (8.38–18)	**< 0.001** ^*^
BUN > 8.4 mmol/L, n (%)	249 (30.6)	186 (25.5)	63 (74.1)	**< 0.001** ^*^
**Inflammatory related factors**				
CRP (mg/L), median (IQR)	36.5(15.3–78.53)	34.3(13.9–71.6)	76.1(34.4–137)	**< 0.001** ^*^
CRP > 70 mg/L, n (%)	233(28.6)	186(25.5)	47(55.3)	**< 0.001** ^*^
PCT (ng/mL), median (IQR)	0.1(0.05–0.27)	0.09(0.05–0.21)	0.38(0.14–1.47)	**< 0.001** ^*^
PCT > 0.2 ng/mL, n (%)	245(30.1)	191(26.2)	54(60.5)	**< 0.001** ^*^
LDH (U/L), median (IQR)	242(199-302.5)	236(194–289)	346(275–459)	**< 0.001** ^*^
LDH > 311 U/L, n (%)	184(22.6)	129(17.7)	55(64.7)	**< 0.001** ^*^
CK-MB (U/L), median (IQR)	15(12–21)	15(12–20)	20(14–28)	**< 0.001** ^*^
CK-MB > 19 U/L, n (%)	275(33.7)	224(30.7)	51(60)	**< 0.001** ^*^
**Blood coagulation factor**				
D-dimer (µg/L), median (IQR)	764(404–1764)	700(380–1491)	2150(1124–7090)	**< 0.001** ^*^
D-imer > 1257 µg/L, n (%)	273(33.5)	211(28.9)	62(72.9)	**< 0.001** ^*^
FIB (g/L), median (IQR)	4.49(3.69–5.17)	4.49(3.73–5.14)	4.51(3.39–5.28)	0.409
FIB > 4.7 g/L, n (%)	349(42.8)	310(42.5)	39(45.9)	0.627
**Cytokines**				
IL-1β (pg/mL), median (IQR)	0.11(0.1–0.39)	0.11(0.1–0.39)	0.11(0.1–0.51)	0.764
IL-1β > 1 pg/mL, n (%)	737(90.4)	664(90.1)	73(85.9)	0.190
IL-5 (pg/mL), median (IQR)	0.92(0.1–2.12)	0.92(0.1–2.17)	0.88(0.01–1.9)	0.495
IL-5 > 0.05 pg/mL, n (%)	177(21.7)	153(21.0)	24(28.2)	0.161
IL-12P70 (pg/mL), median (IQR)	2.34(0.01–4.09)	2.36(0.01–4.07)	2.15(0.01–4.38)	0.931
IL-12P70 > 1.1 pg/mL, n (%)	278(34.1)	243(33.3)	35(41.2)	0.183
IFN-α (pg/mL), median (IQR)	1.44(0.58–2.67)	1.45(0.6–2.67)	1.35(0.42–2.58)	0.642
IFN-α > 0.34 pg/mL, n (%)	140(17.2)	120(16.4)	20(23.5)	0.137
IL-2 (pg/mL), median (IQR)	1.79(0.29–2.73)	1.81(0.26–2.73)	1.65(0.36–2.83)	0.94
IL-2 > 1.7 pg/mL, n (%)	391(48.0)	347(47.5)	44(51.8)	0.533
IL-4 (pg/mL), median (IQR)	2.31(0.01–3.13)	2.33(0.01–3.14)	1.67(0.01–2.97)	0.234
IL-4 > 0.25 pg/mL, n (%)	239(29.3)	206(28.2)	33(38.8)	0.057
IL-6 (pg/mL), median (IQR)	5.72(3.92–12.72)	5.32(3.77–10.8)	17.2(9.75–47.8)	**< 0.001** ^*^
IL-6 > 9.5 pg/mL, n (%)	272(33.4)	207(28.4)	65(76.5)	**< 0.001** ^*^
IL-10 (pg/mL), median (IQR)	3.94(2.32–6.13)	3.76(2.24–5.73)	5.36(3.43–10.7)	**< 0.001** ^*^
IL-10 > 5.1 pg/mL, n (%)	285(35.0)	234(32.1)	51(60.0)	**< 0.001** ^*^
TNF-α (pg/mL), median (IQR)	2.48(0.74–4.4)	2.51(0.742–4.37)	1.94(0.53–4.4)	0.887
TNF-α > 1.3 pg/mL, n (%)	305(37.4)	270(37.0)	35(41.2)	0.524
IFN-γ (pg/mL), median (IQR)	1.91(0.56–3.01)	1.9(0.57–2.97)	2(0.39–3.39)	0.987
IFN-γ > 3.4 pg/mL, n(%)	660(81.0)	596(81.6)	64(75.3)	0.206
IL-17 A (pg/mL), median (IQR)	0.1(0.01–19.69)	0.1(0.01–21.6)	0.1(0.01–7.18)	0.146
IL-17 A < 89 pg/mL, n (%)	797(97.8)	716(98.1)	81(95.3)	0.108

*Notes* *CK-MB* creatine kinase muscle isoenzyme; *CRP* C-reactive protein; *FIB* fibrin/fibrinogen degradation products; *IQR* interquartile range; *LDH* lactate dehydrogenase; *Lym* lymphocytes; *Neu* neutrophils; *NLR* neutrophil-to-lymphocyte ratio; *PCT* procalcitonin; *WBC* white blood cell. Statistical significance is indicated in bold

### Risk factors and the prediction model for death in patients with SARS-CoV-2 Omicron infection

**Fig. 1:**
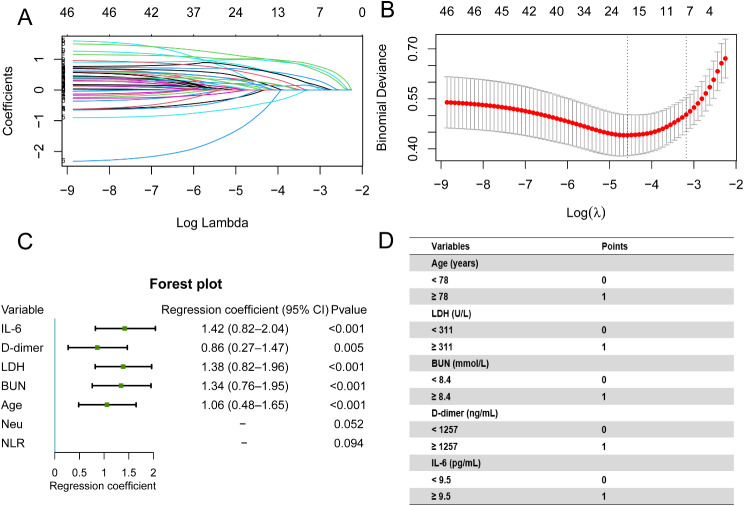
Least absolute shrinkage and selection operator (LASSO) regression and multivariable logistic regression were used to screen clinical features associated with COVID-19 mortality in the training set. *Notes* (**A**) LASSO trace curves of 46 features with < 10% missing values. (**B**) Seven features were selected using LASSO binary logistic regression analysis, and the two dashed vertical lines marked the optimal values using the minimum criteria and the 1 standard error (SE) of the minimum criteria (the 1 − SE criteria). (**C**) Regression coefficients obtained using multivariable logistic regression. (**D**) Scoreing of variables in the scoring system

After excluding the variables with missing values in ≥ 10% of records in the training set, 46 clinical features detected on admission were analysed using LASSO binary logistic regression. All features were categorical variables, and seven factors were significantly associated with the risk of COVID-19 death, including age, IL-6, blood urea nitrogen (BUN), lactate dehydrogenase (LDH), D-dimer, neutrophil count, and neutrophil-to-lymphocyte ratio (NLR) (Fig. 1A and B). Multivariable logistic regression analysis showed that older age and IL-6, LDH, BUN and D dimer levels were independent risk factors for mortality (Fig. 1C). The risk-scoring system was generated by assigning points based on regression coefficients. The score distributions were as follows: age ≥ 78 years, 1 point; IL-6 ≥ 9.5 pg/mL, 1 point; BUN ≥ 8.4 mmol/L, 1 point; LDH ≥ 311U/L, 1 point; and D-dimer ≥ 1257 ng/mL, 1 point (Fig. 1D).

### Performance of the scoring system in the training and independent validation set

**Fig. 2:**
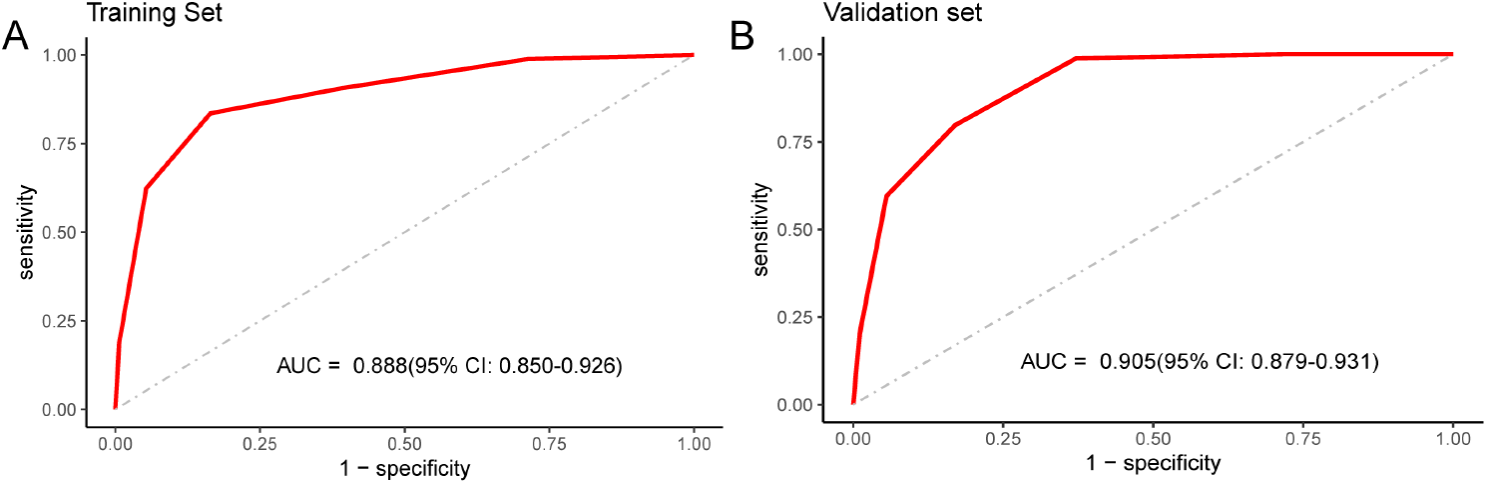
Evaluation of the performance of the scoring system in predicting death in patients hospitalized with SARS-CoV-2 Omicron infection. *Notes* Receiver operating characteristic (ROC) curves and the area under the curve (AUC) were used to evaluate the accuracy of the scoring system in the training set (**A**) and the independent verification set (**B**). *AUC* area under the curve; *CI* confidence interval

To confirm the generalisability of the risk score, we used an independent group of 1,002 patients from seven hospitals in different regions of the country. The same variables were collected from the validation set and the risk scores were calculated. ROC analysis indicated a good predictive performance in both the training set (AUC: 0.888; 95% CI: 0.850–0.926) and the independent validation set (AUC: 0.905; 95% CI: 0.879–0.931) (Fig. 2A and B). The Youden index-based cutoff generated during the development of the scoring system was 2.5, with a 83.6% sensitivity, 83.5% specificity, 37.2% positive predictive value (PPV), and 97.8% negative predictive value (NPV). In the validation set, sensitivity, specificity, PPV, and NPV were 79.8%, 83.0%, 30.0%, and 97.8%, respectively.

## Discussion

Although most cases of COVID-19 are not life-threatening, the mortality rate is higher in older adults with multiple comorbidities [11].The ongoing evolution and mutation of COVID-19 will lead to more reinfections, and understanding of the risk factors for death from COVID-19 will continue to improve. The purpose of developing a mortality prediction scoring system is to assist clinicians in identifying patients at high risk of death on admission when the associated symptoms may be mild and nonspecific.

In this multicentre retrospective study, we developed and validated a new predictive score based on five variables (age and IL-6, BUN, D-dimer, and LDH levels) to predict outcomes in patients with SARS-CoV-2 Omicron infection. Compared with the traditional SOFA (AUC: 0.7) and qSOFA scores (AUC: 0.61), our model has a better ability to predict COVID-19 deaths [12]. The independent validation data were collected from seven hospitals in multiple provinces and cities across the country. Despite differences from the training set, the scoring systems also had high accuracy in the validation set, indicating that the scoring system is generalisable. In addition, some laboratory assays differed by hospital. For example, IL-6 measurement is not as standardised as other inflammatory markers, which also suggests that different laboratory techniques can be used without affecting the model performance.

In our study, the risk factors for death in patients with COVID-19 were similar to those identified in previous studies [13, 14]. Older patients have more underlying diseases, are more likely to have secondary infections, and develop critical illness and have a higher case fatality rate [15]. IL-6 is a multifunctional cytokine that regulates humoral and cellular responses, and has been identified in many previous studies as an important biomarker associated with adverse clinical outcomes of COVID-19 [16, 17]. It is released by immune cells, including macrophages and T cells, and elevated levels of IL-6 reflect viral load and lung damage. Overexpression of proinflammatory cytokines and chemokines is involved in the occurrence of severe pneumonia, acute respiratory distress syndrome, and multiple organ failure in patients with COVID-19 [18]. Our study suggests that monitoring IL-6 levels can also help identify high-risk patients. In our study, elevated D-dimer levels were associated with mortality. D-dimer is a fibrin degradation product. An increase in D-dimer level indicates activation of coagulation, which may be related to thrombosis and inflammation [19]. D-dimer levels are elevated in 3.75–74.6% of patients with COVID-19 [20, 21]. A multicentre retrospective study conducted in Wuhan found that D-dimer greater than 1 µg/mL on admission was associated with an increased risk of in-hospital death [22]. Consistent with previous studies, we found that elevated BUN levels were associated with an increased risk of death from COVID-19 [23]. BUN, a nitrogenous end-product of protein metabolism, can be used to assess renal function and Hypovolemia. One study reported that after correcting for renal function, a high BUN concentration on admission was still closely related to the adverse outcomes of critically ill patients in the ICU [24]. In addition, the BUN-to-serum albumin ratio is an important prognostic factor for mortality and severity in patients with aspiration pneumonia, hospital-acquired pneumonia, and community-acquired pneumonia [25, 26]. LDH is an enzyme present in the cytoplasm that is involved in lactate metabolism. An elevated LDH level is an indicator of cell damage or necrosis [27]. Several studies have shown that the LDH levels reflects disease severity and is significantly higher in patients in ICUs than in other patients [28, 29]. A meta-analysis of 18 studies (total sample size: 5394 patients) showed that an elevated LDH level was associated with a 5-fold increase in the risk of adverse outcomes in patients with COVID-19 [30]. The discovery of these biomarkers also has implications for the treatment of COVID-19, such as timely anti-inflammation, blocking cytokine storm, appropriate anticoagulation, and prevention of gastrointestinal bleeding may help to improve prognosis.

It is worth noting that underlying disease (especially cardiac disease) have been associated with poor prognosis [31], the prediction of COVID-19 death in our study was mainly captured by age and biological examination at adimission. Machine learning variable selection techniques essentially retain only those variables that have the greatest impact on prognosis, and the extent of individual systemic inflammatory response syndrome appears to drive patient outcomes to a greater extent than underlying conditions. Therefore, underlying disease was not included in the final risk score because its effect was offset by other factors.

Our scoring system has several advantages. First, it is based on the data of patients with SARS-CoV-2 Omicron infection, and so adds to the prognostic indicators available for different variants of SARS-CoV-2. Second, it is based on readily available objective indicators, and is easy to calculate and use in clinical practice. Third, it has good prediction performance and was verified using data from different hospitals in China, so has generalisability. However, our research has some limitations. First, this study was retrospective, not all patients underwent all laboratory tests, and all patients in this study were hospitalized and none were outpatients, resulting in incomplete data and selection bias. Second, it is not a large-sample study. All the data come from China and may not fully represent the world ‘s population. Third, our risk score was calculated from baseline variables at admission, regardless of the effect of various treatments during hospitalization on prognosis such as antiviral therapy, which was an important independent predictor of COVID-19-related mortality [32]. In addition, most of the patients in our study received COVID-19 vaccines on national appeal, but we did not collect specific information on the number, type, and timing of vaccinations, which have been reported to reduce the risk of COVID-19-related death in a dose-response manner [33]. These limitations may limit its implementation. Large-scale prospective data are needed to optimize the model in the future.

In summary, the scoring system based on age and four laboratory indicators on admission can timely and effectively assess the risk of patients with SARS-CoV-2 Omicron infection, and help clinicians identify high-risk patients for monitoring and immediate intervention.

### Electronic supplementary material

Below is the link to the electronic supplementary material.

Supplementary Material 1

## Data Availability

No datasets were generated or analysed during the current study.

## Abbreviations

IL: Interleukin
BUN: Blood urea nitrogen
LDH: Lactate dehydrogenase
NLR: Neutrophil-to-lymphocyte ratio
SARS-CoV-2: Severe acute respiratory syndrome coronavirus 2
IQRs: Interquartile ranges
ROC: Receiver operating characteristic
LASSO: Least absolute shrinkage and selection operator
AUC: Area under the curve, CCVD: Cardio-cerebrovascular disease
PPV: Positive predictive value
NPV: Negative predictive value
