# Supplementary material for: Development and validation of a scoring system to predict the mortality of hospitalized patients with SARS-CoV-2 Omicron: a nationwide, multicentre study
Source: BMC Pulm Med. 2024 Jul 3;24:312. doi: 10.1186/s12890-024-03131-5 (PMC11223413; doi:10.1186/s12890-024-03131-5)
Supplement: Supplementary file 1 — Supplementary Material 1 [file 12890_2024_3131_MOESM1_ESM.docx]

Supplementary Table 1 All variable included in analysis in training set

| Variable included in analysis | Cut off point of the First Affiliated Hospital of Zhejiang University School of Medicine |
| --- | --- |
| Age ≥78 y | / |
| Gender | / |
| Days from illness onset to  Hospital admission | / |
| Hypertension | / |
| Diabetes | / |
| Chronic obstructive pulmonary disease | / |
| Cardio-cerebral-vascular disease | / |
| [Chronic liver disease](javascript:;) | / |
| [Chronic kidney disease](javascript:;) | / |
| [Malignant tumor](javascript:;) | / |
| Fever | / |
| Cough | / |
| Dyspnea | / |
| Fatigue | / |
| Nausea/vomiting | / |
| Diarrhea | / |
| White blood cell | (4-10) ×10^9^ /L |
| Neutrophils | (2-7) ×10^9^ /L |
| Lymphocytes | (0.8-4) ×10^9^ /L |
| Platelet | (83-303) ×10^9^ /L |
| Neutrophil-to-lymphocyte ratio | / |
| C-reactive protein | 8 mg/L |
| Procalcitonin | 0.5 ng/mL |
| Alanine transaminase | 7-40 U/L |
| Aspartate transaminase | 13-35 U/L |
| Total bilirubin | 21 umol/L |
| Direct bilirubin | 8 umol/L |
| Albumin | 40-55 g/L |
| Blood urea nitrogen | 3.1-8.8 mmol/L |
| Creatinine | 41-81 umol/L |
| Triglyceride | 0.3-1.7 mmol/L |
| Lactate dehydrogenase | 120-250 U/L |
| Creatine kinase muscle isoenzyme | 2-25 U/L |
| D-dimer | 700 ug/L |
| Fibrin/fibrinogen degradation products | 2-4 g/L |
| Interleukin-1β | 12.4 pg/mL |
| Interleukin-5 | 3.1 pg/mL |
| Interleukin-12P70 | 3.4 pg/mL |
| IFN-α | 8.5 pg/mL |
| Interleukin-2 | 5.71 pg/mL |
| Interleukin-4 | 3 pg/mL |
| Interleukin-6 | 5.3 pg/mL |
| Interleukin-10 | 4.91 pg/mL |
| TNF-α | 4.6 pg/mL |
| IFN-γ | 7.42 pg/mL |
| Interleukin-17A | 20.6 pg/mL |

Supplementary Table 2 Cut off point of each center included in validation set

|  | Shenzhen Third People's Hospital | Affiliated Dongyang Hospital of Wenzhou Medical University | Shulan Hospital of Hangzhou | Fifth Medical Center of People’s Liberation Army General Hospital | Beijing Ditan Hospital Affiliated to Capital Medical University | Qilu Hospital of Shandong University | The First Affiliated Hospital of China Medical University |
| --- | --- | --- | --- | --- | --- | --- | --- |
| Interleukin-6 | 7 pg/ml | 5.4 pg/ml | 5.4 pg/ml | 7 pg/ml | 7 pg/ml | 5.30 pg/ml | 5.30 pg/ml |
| Blood urea nitrogen | 3.1-8 mmol/L | 2.6-7.5 mmol/L | 3.1-8 mmol/L | 2.9-8.2 mmol/L | 3.1-8 mmol/L | 2.3-7.8 mmol/L | 3.2-7.1 mmol/L |
| D-dimer | 500 ug/L | 500 ug/L | 243 ug/L | 550 ug/L | 500 ug/L | 500 ug/L | 500 ug/L |
| Lactate dehydrogenase | 120-250 U/L | 120-250 U/L | 120-250 U/L | 109-245U/L | 120-250U/L | 120-230 U/L | 120-246 U/L |
